# A four-year clinical and sonographic longitudinal follow-up of clubfeet treated according to Ponseti with normal references

**DOI:** 10.1177/18632521231172548

**Published:** 2023-05-17

**Authors:** G Arne Johansson, Ylva B Aurell, Bertil H Romanus

**Affiliations:** 1 Department of Orthopaedics, Institute of Clinical Sciences, Sahlgrenska Academy, University of Gothenburg, Gothenburg, Sweden; 2 Department of Orthopaedics, Region Västra Götaland, Skaraborg Hospital, Skövde, Sweden; 3 Department of Radiology, Institute of Clinical Sciences, Sahlgrenska Academy, University of Gothenburg, Gothenburg, Sweden; 4 Department of Diagnostic Radiology, Region Västra Götaland, Sahlgrenska University Hospital, Gothenburg, Sweden

**Keywords:** Clubfoot, ultrasonography, Ponseti treatment, congenital deformity, foot deformity

## Abstract

**Purpose:**

To follow children with a clubfoot by ultrasonography during the entire treatment period up to 4 years and compare with controls.

**Method:**

Thirty clubfeet in 20 children treated using the Ponseti method and 29 controls were followed by repeated ultrasonography investigations from neonates to the age of 4 years. The previously established coronal medial and lateral, sagittal dorsal and posterior projections were used. Changes over time, correlations to the Diméglio score, and the course of treatment were studied.

**Results:**

The medial malleolus–navicular distance was shorter, while the talar tangent–navicular distance and the talo-navicular angle were larger in clubfeet than in controls even after the initial correction. The healthy feet in unilateral cases did not differ significantly from the controls. The range of motion in the talo-navicular joint was approximately 20° less in clubfeet than in controls during the first four years of life. The medial malleolus–navicular distance (*r* = –0.58) and the talo-navicular angle (*r* = 0.66) at the first ultrasonography showed the highest correlation to the number of casts needed to correct the deformities.

**Conclusion:**

Ultrasonography can be used to evaluate the initial degree of deformities in clubfeet and to follow the progress of the treatment and growth. Ultrasonography showed a clear difference between clubfeet and controls during the first four years of life. Although it was not possible to define specific limit values as benchmarks in the treatment, dynamic ultrasonography can provide valuable support in the decision-making process when complementary treatment may be needed.

**Level of evidence:**

III

## Background

Clubfoot is a common congenital deformity with a geographical variation in prevalence from 1 to 6 out of 1000 live births.^1–4^ The non-surgical Ponseti method is currently widely accepted as the treatment of choice.^1^ The treatment starts with manipulations and castings (the correction phase). Before the application of the last cast, a percutaneous Achilles tendon tenotomy (ATT) is needed in approximately 80% of the feet. The correction phase is followed by treatment with a foot abduction orthosis (FAO) until the age of 4–5 years (conservation phase). Only a few percentage require complementary surgery by posterior or posteromedial release to achieve the initial correction of the deformities.^5^ At a later stage of treatment (usually 3–5 years of age), anterior tibial tendon transfer is performed on the indication of supination or recurrence in a sizable percentage of the clubfeet; Ponseti and Smoley^6^ reported 42%, while Laaveg and Ponseti^7^ reported 46%. The orthosis treatment to the age of four years of age is essential to prevent relapses,^8–10^ but relapses can occur up to skeletal maturation. A few percentage of the clubfeet are atypical in terms of the clinical appearance: short and stubby, severe equinus with a deep crease above the heel, short calf muscles, high cavus with a deep crease across the full width of the sole and hyperextended big toe.^11,12^ They are usually more difficult to treat and require a modified casting technique.^11,12^ The two terms “atypical” and “complex” are often used as though they were synonyms.^13^ In Ponseti International Association (PIA) guidelines, the term “atypical” is used for feet with this appearance before the start of treatment, while the term “complex” is used for feet acquiring this appearance during the casting treatment.^14^ Complex clubfeet have the same feature as the atypical ones, as well as edema, redness, and hyperesthesia.^15^ Other authors making the same distinction are Al-Mohrej et al.,^16^ Allende et al.,^17^ and Duman et al.^18^ Duman et al. and Dragoni M. et al. named the latter group “iatrogenic complex clubfeet.” Dragoni et al.^15^ found that all feet with slipping casts were not converted from typical to complex clubfeet, but some were, probably because of a combination of some intrinsic predisposing factors (high Pirani score, short and stubby clubfoot, partly fibrotic muscles in the plantar compartment, and a short triceps surae) and slipping casts.

### Imaging

During infancy, when the treatment takes place, conventional radiography (CR) provides limited information about the foot skeleton because the skeleton is only partly ossified. Magnetic resonance imaging (MRI) visualizes cartilaginous parts of the skeleton, as well as soft tissues, but it requires sedation.^19^ Ultrasonography (US) is a dynamic radiation-free image modality suitable for imaging non-ossified parts of the skeleton in infant feet.^20–24^ US protocols with standard projections and reliable variables for assessing normal feet and clubfeet during the first years of life have been presented.^20,25–28^ Furthermore, Bhargava et al.^24^ found that US evaluation had a better correlation to the severity scoring of clubfeet than CR. The previous studies have been cross-sectional studies or have focused on the first year of life.^26,27,29^ After the initial correction phase, the treatment with an orthosis continues to the age of four years, a prerequisite to prevent recurrence.^8,9^ To the best of our knowledge, no studies have been published in which the feet have been followed by repeated US scans during the whole treatment period from the start of treatment in the neonatal period to the age of 4. The individual variation in growth and development is considerable and this is not revealed in cross-sectional studies. In this study, the same individuals are followed by repeated dynamic US investigations to study the changes in the anatomical structures during the entire treatment, to be able better to understand the growth and development of clubfeet as well as normal feet.

## Purpose

The purpose was

- To prospectively follow and document the treatment of clubfeet from the initial casting treatment through the brace treatment to the age of 4 years using repeated US.
- To compare the development of the clubfeet during treatment with the development in controls.
- To study the clinical development of the clubfeet and the need for complementary treatment, before and after the termination of the orthosis treatment.

## Materials

There were 22 consecutive children born between 2005 and 2011, treated for clubfoot according to the Ponseti method at the Department of Orthopedics at a county hospital. During this period, the clubfeet treated at the clinic routinely underwent repeated US at fixed time intervals during the first four years of life. Of these, one was excluded due to a congenital syndrome affecting the feet and one had emigrated and was lost to follow-up. The remaining 20 children, 14 boys and 6 girls, were included in the study, 10 had bilateral and 10 had unilateral clubfoot, that is, 30 clubfeet and 10 contralateral normal feet were included. The 10 contralateral normal feet were not included in the control group but were assessed separately, forming a separate group because they could possibly be influenced by the bilateral FAO. Two of the clubfeet had signs of atypical deformities before starting treatment and another four became complex during the treatment due to slipping casts. In this study, the term “atypical” is used for feet with atypical signs before starting treatment and “complex” for feet acquiring this appearance during the casting treatment.

A control group of 29 healthy children (18 boys and 11 girls, 58 feet) were recruited from the maternity departments at the same county hospital and a university hospital. Four of the controls discontinued at the age of 2.5 to 4 years.

## Methods

The clubfeet were all treated according to the Ponseti method, which was routine at the clinic. The manipulation and casting treatment (correction phase) started as early as possible with the aim of achieving 60° of abduction and 10°–15° of dorsiflexion. If dorsiflexion of ≥ 10° in the ankle joint was not achieved, a percutaneous tenotomy of the Achilles tendon was performed before application of the last cast(s). If the above was not sufficient to correct the deformities, the treatment was completed by soft-tissue release, preferably posterior release or lengthening of the posterior tibial tendon. After the initial correction by casting, the treatment continued by orthosis, during the first 3 months, 23 h/day, thereafter during nights and naps, for at least 10–12 h/day, to the age of 4 years. The first choice of orthosis was an FAO and the second choice was a knee–ankle–foot orthosis (KAFO) if the child was unable to tolerate the FAO. Transfer of the anterior tibial tendon was performed on the indication of dynamic supination or as part of the treatment of recurrence. Recurrences were primarily treated by casting, physiotherapy, and orthosis and secondarily by surgery. The atypical and complex clubfeet were treated using the modified Ponseti method with 60° supination of the foot in the first cast and 120° flexion of the knee. These feet were corrected to 40° of abduction and the orthoses were set at 40° of abduction.

The first US was performed before the start of treatment if possible (20 clubfeet in 13 children) or else at one of the first plaster changes (10 clubfeet in seven children). Ultrasound investigations were then repeated at the ages of 3, 6, 12, 18, 24, 30, 36, 42, and 48 months. The contralateral normal feet in unilateral cases were examined on the same occasions as the clubfeet. The control feet were investigated at the same ages. The child’s foot was held in the standardized positions by the treating orthopedic surgeon during the ultrasound scanning. The dynamic ultrasound scans were performed by one of four radiologists who were experienced US examiners. As the treating physician performed the Ponseti maneuvers during the US investigation, it was impossible to stay 100% blinded to the imaging content, although the intention was to follow the current routine at the clinic.

The examinations were performed with a high-frequency linear transducer (8 MHz to 15 MHz and 5 MHz to 17 MHz). Three different US machines were used: Acuson, Sequoia (Acuson, Mountain View, California), Philips iU22 (Amsterdam, Netherlands), and GE Logiq E9 (Chicago, Illinois, USA).

Frozen still images were stored in the regional radiological archive and measurements were performed using Picture and Archiving System (PACS) software (Centricity PACS, GE Healthcare Integrated IT Solutions, Barrington, IL, and SECTRA PACS, Linköping, Sweden).

The medical records were reviewed from the first visit to the orthopedic clinic to the age of 8 years. The initial Diméglio score (routinely used at the clinic)^30^ and the course of treatment: number of casts, tenotomies of the Achilles tendon, orthosis treatment, relapses, and complementary treatment to the age of 8 years were recorded.

### Projections and foot positions

Four previously established repeatability tested projections were used: coronal medial, coronal lateral, sagittal dorsal, and sagittal posterior.^25–28^ The coronal medial and lateral investigations were performed with the foot in adducted, neutral, and abducted positions, according to the protocol described by Johansson et al.^27^ The sagittal dorsal projection was performed with the foot in slightly plantar flexed position.^27^ The sagittal posterior projection was performed with the foot maximally plantar flexed, in a neutral position (or as near neutral as possible in untreated clubfeet) and in maximal dorsiflexion (after the correction of the equinus deformity in clubfeet), according to the protocol presented by Johansson et al.^28^

### Measurements

Previously established intra- and inter-observer repeatability tested variables were used.^25–29^ All the 11 variables used presented below were described and defined by Johansson et al.^27,28^ in two articles (distributed under CC BY-NC 4.0 license) and in one of the author’s (AJ) dissertation.^31^ The definitions of the variables in figure legends 1–6 are from the same publications. Each variable was measured once by the pediatric orthopedic surgeon (AJ) several months after the imaging and without access to the medical records.

#### At the coronal medial projection

**Figure 1.**
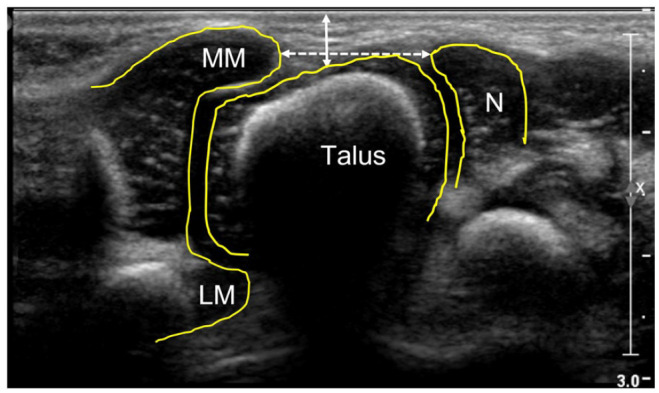
Coronal medial US scan of a normal foot in a newborn boy. MM: medial malleolus; LM: lateral malleolus; N: navicular bone; solid arrow: soft tissue thickness (STT); dashed arrow: medial malleolus–navicular (MM-N) distance.

**Figure 2.**
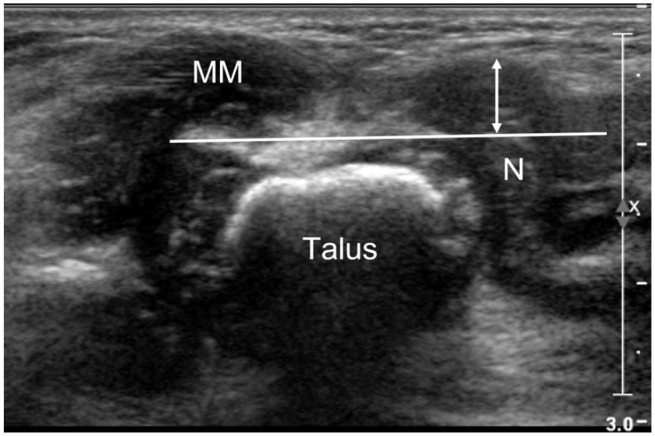
Coronal medial US scan of a clubfoot in a 6-month-old boy. Double arrow represents the talar-tangent–navicular (T-Tang-N) distance, the perpendicular distance from the medial tangent of the talus to the medial border of the navicular. The values were defined as positive (+) when the medial border of the navicular was medial to the talar tangent and negative (–) when it was lateral to the tangent. MM: medial malleolus; N: navicular bone.

**Figure 3.**
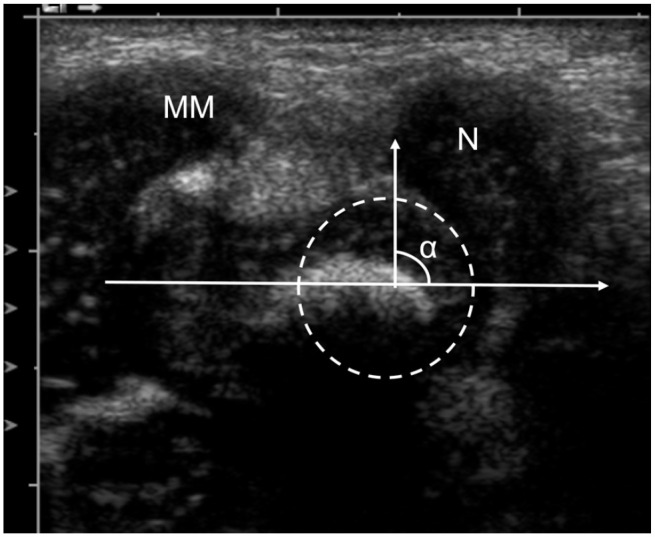
Coronal medial US scan of a clubfoot in a 1-month-old girl. α is the talo–navicular (T-N) angle, the angle between the longitudinal axis of the talus and a line drawn from the medial corner of the navicular to the center of the talar head. The center of the talar head was determined using the “region of interest (ROI)” tool in the PACS. The size-adjustable circle was laid over the periphery of the talar head and the lines were drawn through the marked midpoint. MM: medial malleolus; N: navicular bone.

- Soft tissue thickness (STT; Figure 1)
- Medial malleolus–navicular (MM-N) distance (Figure 1)
- Talar tangent–navicular (T-Tang-N) distance (Figure 2)
- Visual semi-quantitative grading of the medial displacement of the navicular bone (normal, subluxated, or luxated)
- talo–navicular (T-N) angle (Figure 3)
- The range of motion (ROM) in the T-N joint in the coronal plane was calculated as the difference between the T-N angle in maximally adducted and maximally abducted positions of the foot (also see supplementary material Video 1).

#### At the sagittal dorsal projection

**Figure 4.**
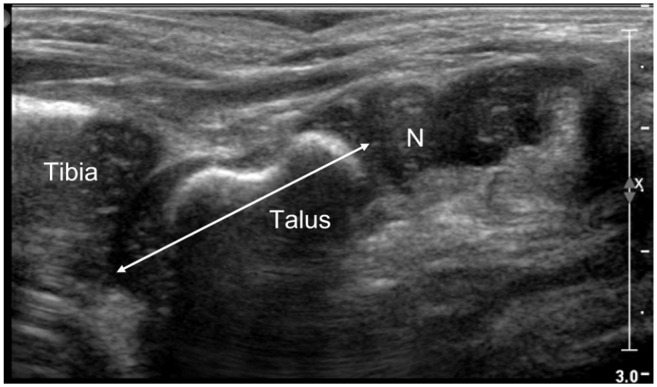
Sagittal dorsal US scan of a normal foot in a 6-week-old boy. Double arrow represents the length of the talus. Normal position of the navicular (N)

- Length of the talus (Figure 4)
- The position of the navicular bone was evaluated as normal, dorsally, or plantarly displaced in relation to the head of the talus.

#### At the coronal lateral projection

**Figure 5.**
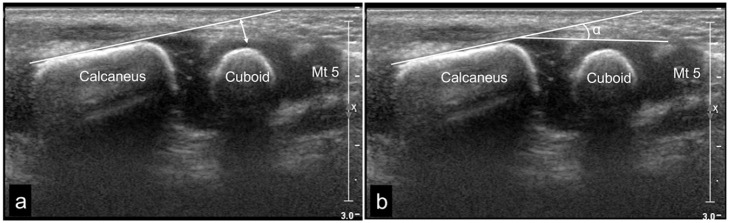
Coronal lateral US scan of the normal right foot in neutral position in a 3-week-old boy: (a) Calcaneo-cuboid (C-C) distance, the perpendicular distance from the lateral tangent of the calcaneus to the middle of the lateral border of the cuboid. (b) Calcaneo-cuboid (C-C) angle, the angle between the tangents of the lateral borders of the calcaneus and the cuboid. The values were defined as positive (+) when the angle was medially open and negative (–) when the angle was laterally open related to the tangent of the calcaneus. Mt 5 represents the proximal metaphysis of the fifth metatarsal bone.

- Calcaneo-cuboid (C-C) distance (Figure 5(a))
- Calcaneo-cuboid (C-C) angle (Figure 5(b))

#### At the sagittal posterior projection

**Figure 6.**
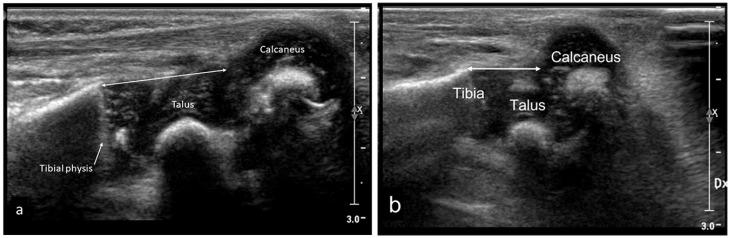
Sagittal posterior US scan. (a) The left normal foot in a neutral position in a 7-week-old girl. Double arrow represents tibial physis to the posterior border of the talo-calcaneal joint distance (Tib. phys.-TCJ). (b) The right-sided clubfoot at the first plaster change in a 14-day-old girl. Double arrow represents the tibial physis–calcaneal distance (Tib. phys.-C), the shortest distance from the posterior border of the tibial physis to the cranial surface of the calcaneus. Note the anterior position of the talus.

- The tibial physis–talo calcaneal joint (Tib. phys.-TCJ) distance was measured with the foot in a neutral position and in maximum dorsiflexion (Figure 6(a)).
- The tibial physis–calcaneal (Tib. phys.-C) distance was used for measurements with the foot in a plantar flexed position and for clubfeet with remaining equinus (Figure 6(b)).

### Statistical analyses

Descriptive statistics were presented as the mean with 95% CI (confidence interval) or the median with quartiles for continuous variables and as frequencies and percentages for categorical variables. As some of the statistical comparisons dealt with small groups or non-normally distributed data, we used non-parametric tests (Mann–Whitney) for comparisons with respect to continuous variables and Spearman’s correlation for exploring bivariate correlations between different continuous variables. Wilcoxon’s test was used for correlations within group. All calculations were made using IBM SPSS Statistics 25. Correlation interpretation: 0.9–1 very high, 0.7–0.9 high, 0.5–0.7 moderate, 0.3–0.5 low, 0.0–0.3 negligible.^32^

In cases with bilateral clubfeet, both feet were included in the statistical calculations and in the charts, unless otherwise stated. As the data about 2 feet from the same patient cannot be regarded as independent observations, it would be statistically correct only to keep one foot in the analysis for the bilateral cases. We therefore performed a sensitivity analysis with only one foot from bilateral cases to explore whether the bilateral cases affected the results. In the sensitivity analysis, we tested the correlation between the number of casts and the US variables of MM-N distance, T-Tang-N distance and T-N angle at the first US examination, with only one of the feet in bilateral cases included, while every other right or left foot was included in the order they appeared in the participant list.

## Results

### Overall course of treatment

All 30 clubfeet were treated by manipulations and castings according to the Ponseti method. After excluding two complex outliers, the mean number of casts was 6.8 (range 2–12). The two outliers had 34 and 40 casts, respectively and both had a posterior release combined with lengthening of the posterior tibial tendon during cast treatment.

Twenty-one feet had a percutaneous Achilles tenotomy (ATT); of these, three (in two children) had a second tenotomy during the casting treatment.

After the casting, 29 clubfeet primarily received an FAO, one received a KAFO, later changed to an FAO due to difficulty achieving sufficient abduction and the child not being comfortable with the KAFO. Five were changed from an FAO to a KAFO due to sleeping problems, one was changed from an FAO to an AFO (ankle–foot orthosis) at 3.5 years of age, due to knee and sleeping problems. Fifteen families/children (20 clubfeet) adhered to the brace protocol to the age of 4 years, eight of these children (11 clubfeet) continued to 4.1–5 years. Four children (eight clubfeet) stopped using the brace between 3 and 4 years and one child with mild bilateral clubfeet stopped at 1.5 years.

In addition to the primary ATT during the casting treatment, secondary surgery was needed in 12 feet (eight children, six boys, and two girls), where 5 of these 12 feet were atypical or complex (Tables 1 and 2, master table in supplementary material). The atypical/complex clubfeet also tended to be outliers in several US variables. After the initial casting treatment, four feet had an Achilles lengthening (one ATT and three fractioned percutaneous tenotomy), combined with an anterior tibial transfer due to a relapse. Two ATTs were performed because of a relapse. Six anterior tibial transfers were performed on the indication of supination during gait. Four feet were operated on more than once (for details, see Table 1 and the more extensive Table 2 in supplementary material).

**Table 1.** Complementary surgical procedures, in addition to tenotomies of the Achilles tendon, performed during the casting treatment.

Child no. right (R)/left (L)	Atypical/complex	ATT	Fr.ATT	PR	P.Tib.L	A.Tib.Tr.	A.Tib.Tenodesis
02 R			4 y 5 m				
02 L		2 y	4 y 5 m 6 y 11 m			6 y 11 m	
07 R						8 y 9 m	
07 L	Atypical					8 y 9 m	
10 L		3 y 10 m				3 y 10 m	
12 R		8 m					
13 R						4 y 8 m	6 y 8 m
13 L						4 y 8 m	
15 R	Atypical		4 y 10 m			4 y 10 m	
15 L	Complex		4 y 10 m	4 m	4 m	4 y 10 m	
17 L						7 y 5 m	
20 R	Complex			7 m	7 m	4 y 7 m	
Summary (*n*)	4	3	5	2	2	10	1

In the table, the surgical procedures are represented by the age at which they were performed (y represents years; m represents months). Note that 4 of the 12 operated feet had atypical/complex signs.

Atypical: the feet showed signs of atypical clubfeet before treatment; complex: the feet developed an atypical appearance during the plaster treatment; ATT: percutaneous Achilles tendon tenotomy; Fr.ATT: fractional percutaneous Achilles tendon tenotomy; PR: posterior release; P.Tib.L: posterior tibial tendon lengthening; A.Tib.Tr: anterior tibial tendon transfer; A.Tib.Tenodesis: anterior tibial tenodesis.

### Ultrasonography

Nineteen of 20 patients (28 clubfeet) participated in the US investigations until 4 years of age. One patient (two clubfeet) participated in the US investigations until 18 months of age.

All 29 controls (58 feet) participated until 18 months of age and 22 controls (44 feet) participated until 4 years of age. Two controls (four feet) discontinued after 1.5 years, three (six feet) after 3 years and two (four feet) after 3.5 years.

Sometimes, the children did not co-operate, resulting in an incomplete set of images. The coronal medial image in the neutral position was available in 95.8% at the first US, in 97.9% at 18 months, and in 100% at 4 years. The coronal medial image in the abducted position was available in 95.8% at the first US, in 95.6% at 18 months, and in 98.7% at 4 years (for details, see the complete master table in supplementary material, Table 3).

### Coronal medial projection

#### Soft tissue thickness STT

**Figure 7.**
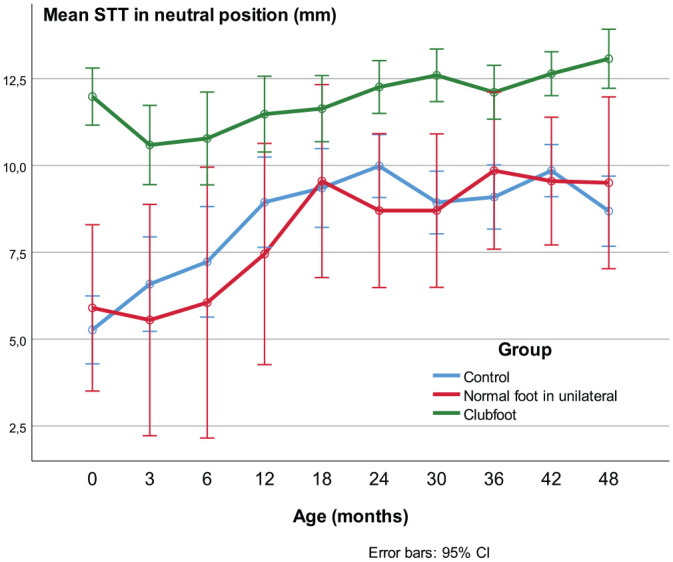
Soft tissue thickness (STT). During the first 3 months, the STT in clubfeet decreased due to the casting treatment. It then increased to the age of 48 months, but in the controls and the normal feet in unilateral cases, the increase flattens out from the age of 18 months.

In clubfeet, the STT decreased from the first US before treatment, during the correction phase, to 3 months of age (*p* = 0.011), but, in controls it increased during this age period (*p* < 0.001; Figure 7). In the controls and the contralateral feet, the increase followed a less steep curve from the age of 1.5 years, while in clubfeet the increase continued at an undiminished rate and, from 1.5 to 4 years, the increase was greater in clubfeet compared with controls (*p* < 0.001). Despite the above-mentioned variation, the STT was greater in clubfeet than in controls and contralateral normal feet at all ages from birth to the age of 4 years (*p* < 0.001 and *p* ≤ 0.02, respectively).

#### MM-N distance

**Figure 8.**
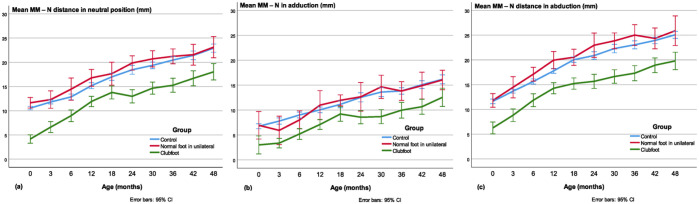
Medial malleolus–navicular (MM-N) distance in the (a) neutral, (b) adducted, and (c) abducted position. In the adducted position, the difference between clubfeet and normal feet is not so large, but in the abducted position the difference is more obvious.

The mean MM-N distance was shorter in clubfeet than in controls and contralateral normal feet in the neutral and abducted position in all age groups (*p* < 0.001; Figure 8(a) and (c)). This was also true in the adducted position, compared with controls (*p* < 0.001) and contralateral normal feet (*p* < 0.05; Figure 8(b)). The ratio between the MM-N in the neutral position and the length of the talus was smaller in the clubfeet than in the healthy controls (*p* < 0.05) in all age groups except at 3.5 years (*p* < 0.07). Moreover, in the abducted position, the ratio between MM-N and talar length was smaller in the clubfeet than in the healthy controls, statistically significant (*p* < 0.05) except at 3, 6, and 12 months of age, but the number of valid observations was smaller in these three age groups, *n* = 15–19, compared with 24–25 in the other groups.

#### T-Tang-N distance

**Figure 9.**
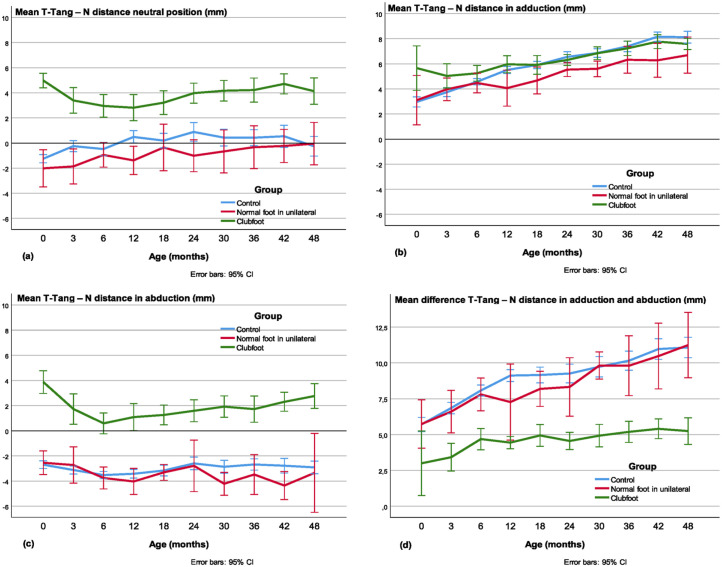
The talar-tangent–navicular (T-Tang-N) distance in the (a) neutral, (b) adducted, and (c) abducted position. In the neutral position, the controls and the normal foot in unilateral cases have values close to zero (i.e. the medial border of the navicular is in line with the medial side of the talus), while the clubfeet have positive values (the navicular is located more medially). Note that, in adduction, there is very little difference between clubfeet and normal feet and, in abduction, the normal feet have negative values, while the clubfeet have positive values. (d) The fourth diagram illustrates the difference between the adducted and abducted position, revealing less mobility in the talo–navicular joint (ROM) in the clubfeet.

The mean T-Tang-N distance was larger in clubfeet than in the controls and the contralateral feet from newborn to the age of 4 years in the neutral (*p* < 0.001; Figure 9(a)) and abducted position (*p* < 0.01; Figure 9(c)). In adduction, the difference was small and statistically significant only at some ages (Figure 9(b)). The difference between the adducted and abducted position (ROM) was less in clubfeet in all investigations compared with the controls (*p* < 0.001). Compared with the contralateral normal foot, the difference in ROM was almost significant at the first investigation and significant at all other investigations (*p* < 0.01), that is, the mobility was less in clubfeet (Figure 9(d)).

#### T-N angle

**Figure 10.**
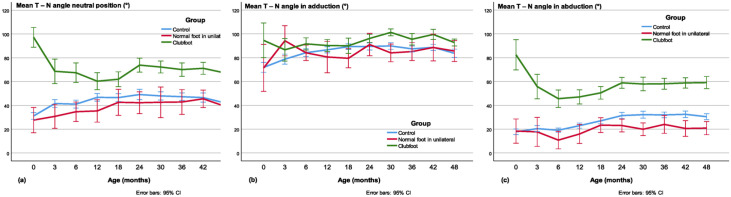
The talo–navicular (T-N) angle in the (a) neutral, (b) adducted, and (c) abducted position. In adduction, there is very little difference between clubfeet and normal feet in all age groups. In the abducted position, the angle was larger in clubfeet, especially before and during the correction phase of the treatment.

The mean T-N angle was larger in clubfeet from newborn to the age of 4 years in the neutral position (Figure 10(a)) compared with controls (*p* < 0.001, except at 12 months *p* = 0.002) and contralateral normal feet (*p* ≤ 0.019) and in abduction (Figure 10(c)) versus controls (*p* < 0.001) and contralateral normal (*p* ≤ 0.005). In adduction (Figure 10(b)), the T-N angle was approximately equal in all three groups.

#### The ROM in the T-N joint

**Figure 11.**
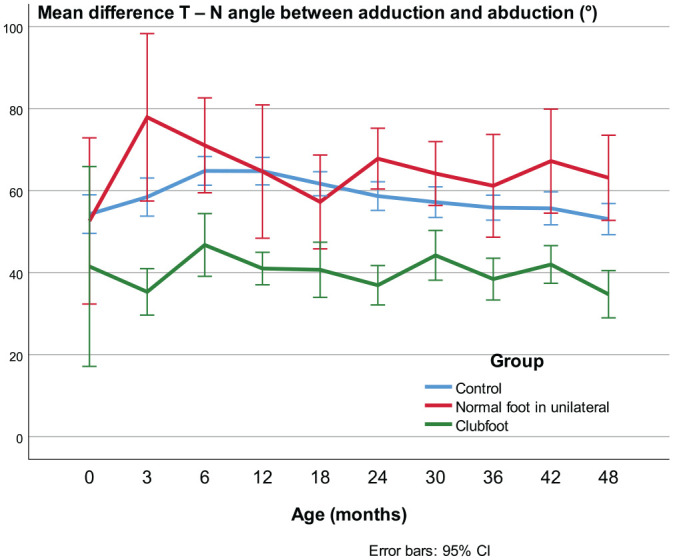
ROM in the talo-navicular joint expressed as the difference between the T-N angle in adduction and abduction.

In newborns to the age of 4 years the ROM in the talo-navicular joint expressed as the T-N angle difference between the adduced and abducted position was approximately 20° less in clubfeet compared with controls (*p* < 0.001) and, compared with the contralateral feet, it was significant (*p* < 0.01), except at the first US investigation before treatment (Figure 11).

### Coronal lateral projection

The C-C distance and the C-C angle were measured, but the measured values and the differences between the adducted and abducted position were small. Despite this, the difference between the C-C distance in the adducted and abducted position was statistically significantly (*p* < 0.05) greater in the healthy controls than in the clubfeet in all age groups except in newborns and at 42 months of age.

### Sagittal dorsal projection

#### Length of the talus

The talus was shorter in clubfeet than in controls.

The mean increase in talar length (growth) from birth to the age of 4 years expressed as the increase in millimeters was larger in controls than in clubfeet (*p* = 0.003), but the difference between the clubfeet and the contralateral normal feet was not statistically significant. Expressed as a percentage of the initial talar length, the increase was larger in clubfeet compared with controls and contralateral normal feet and it was statistically significant from the age of 1 year (*p* < 0.05).

#### Position of the navicular

At the first US investigation of the clubfeet, the navicular had a plantar dislocation in 16 out of 30 feet, a normal position in 11 and data missing for 3. By the age of 1 year, they had all normalized, except for three complex feet which needed another 6 months to normalize.

### Sagittal posterior projection

Of the 19 clubfeet with available Tib. phys.-C data at the first US investigation, the 10 who subsequently needed an achillotenotomy had a shorter Tib. phys.-C distance than the 9 that did not require a tenotomy (*p* < 0.001 according to the Mann–Whitney test). The mean Tib. phys.-C distance was 8.7 and 13.7 mm, respectively.

### Correlation between US and clinical measurements

The Tib. phys.-TCJ distance and dorsiflexion correlation was *r* = 0.35 (*p* = 0.003) at 2 years of age and *r* = 0.2 (*p* = 0.012) at 4 years of age.

### Correlations between the initial US and the Diméglio score before treatment

The T-Tang-N distance in the abducted position was significantly larger (*p* = 0.004) in clubfeet with a Diméglio adductus variable grade 3 compared with those with grade 2 (there was only one grade 1 and no grade 4).

The length of the talus had a negative correlation to the total Diméglio points, *r* = –0.45 (*p* = 0.027). In other words, those with a high Diméglio score had a short talus.

### Correlations between the initial US and the course of treatment

With both feet included in bilateral cases, the correlation between the ultrasound findings at the first US investigation of the newborn patients during the Ponseti maneuver (abducted position) and the number of casts needed to correct the deformities (including two complex outliers, who needed a significantly larger number of casts than the other two complex clubfeet) were negative for the MM-N, *r* = –0.58 (*p* = 0.001), and positive for the T-Tang-N distance, *r* = 0.50 (*p* = 0.01), and the T-N angle, *r* = 0.66 (*p* < 0.001). Without the two complex outliers, the results were consistent.

With only one foot included in bilateral cases, a total of 20 clubfeet, the correlations were *r* = –0.49 (*p* = 0.038 for the MM-N), *r* = 0.60 (*p* = 0.008) for the T-Tang–N distance and *r* = 0.73 (*p* < 0.001) for the T-N angle. These results are consistent with our primary analysis above.

A negative correlation between the MM-N and the number of casts needed as a newborn to correct the deformity remained and was *r* = –0.53 (*p* = 0.004) at the age of 1.5 years and *r* = –0.64 (*p* < 0.01) at the age of 4 years.

## Discussion

According to this study, the medial projection performed prior to or during early treatment is the most useful US approach to forecast the number of casts needed to correct the clubfoot deformity and follow the progress of correction, that is, MM-N, T-Tang-N distance, T-N angle. In atypical/complex clubfeet, the dorsal projection reveals the plantar dislocation of the navicular, causing the severe cavus deformity requiring a modified casting technique.^11^

In the posterior projection, there was a correlation between the Tib. phys.-C distance at the initial US and the need for achillotenotomy. As a result, a short Tib. phys.-C distance indicates that a tenotomy will be needed during the treatment, but a length measurement on US images is influenced by foot size, which must be considered when deciding on a tenotomy. When it is time to decide on a tenotomy, a dynamic posterior sagittal US can be helpful in showing whether the backward rotation of the trochlea tali in the ankle mortis is significantly restricted.

In two complex cases, a posteromedial release was considered, but the dynamic US strengthened the argument to perform only a posterior release.

The ultrasound images were also useful in explaining the deformities, the treatment principles, and the course of treatment to the parents, which may have contributed to good compliance with the orthosis treatment.

It is outside the aim of this study to state the time intervals at which US should be used in a clinical setting, but it might be useful to perform US on demand if there are specific questions. However, this approach requires the US examiner to be familiar with the US technique.

Two complex clubfeet turned out to be outliers in a comparison between the number of casts needed to correct the deformities and the US variables of MM-N, T-Tang-N distance and T-N angle before treatment. The large numbers of castings in the two outliers were largely caused by slipping casts, resulting in frequent plaster replacement. Furthermore, the main difference between atypical/complex clubfeet and other clubfeet is that the former feet have a severe cavus deformity, complicating the treatment. This is not revealed in the medial projection plane but is visualized in the dorsal projection.

The C-C distance and the C-C angle were measured, but the values were small and the measurement errors were, therefore, proportionally large. As a result, the clinical value of these measurements is limited.

Hypoplasia of the talus demonstrated on US has been reported by Chandrakanth et al.^33^ In this study, we found that the percentage growth of the talus was larger in clubfeet than in healthy controls and contralateral normal feet in unilateral cases. Segev et al.^34^ demonstrated by radiography that the size difference in the talus in clubfeet compared with contralateral normal feet decreased with age in well-corrected clubfeet, indicating accelerated growth after the correction of the deformities. The Diméglio score was routinely used at the clinic when the patients were treated. It has subsequently been replaced by the Pirani score, which was considered easier to use. We found a low correlation between the length of the talus and the total Diméglio score. Josse et al.^35^ reported a poor correlation between a modified Diméglio score and a three-grade US score based on the “TMCA formed by the line tangent of the tibial physis and the line tangent to the ossification centres of the talus and calcaneus” and the “TNA subtended by the line tangent of the ossification centre of the talus and the line perpendicular to the navicular bone.” It should be noted that the TN angle used by Josse et al.^35^ and Moritani et al.^36^ is not identical to the T-N angle measurements in this study. Some anatomical abnormalities remained in clubfeet after treatment, which is in accordance with the findings in CR studies.^1,37^

In several of the clinically corrected clubfeet, the navicular was still more medially positioned than normal, indicating that the correction in the T-N joint was not complete. This is sometimes compensated for by a false correction distal to the navicular, which is in accordance with other reports.^1,38^ Adding some scans with a longer probe visualizing the ankle mortis, the talus, the cuneiforms, and the first metatarsal in the same image might reveal these phenomena.

A dorsal scan with a long probe including one of the metatarsals in the image would reveal whether the whole cavus deformity is in the T-N joint or if part of it is more distal. This would be especially interesting in atypical and complex clubfeet. The atypical/complex clubfeet showed a tendency to be more severe in many of the measured parameters and further studies with more material are needed to describe the atypical and complex deformities more thoroughly in these feet.

The children did not always co-operate, making it sometimes impossible to perform a complete US investigation, and this was the main reason for some missing data. It is very important that the child is relaxed during the examination; otherwise, the position and mobility of the joints cannot be depicted correctly (Video 1). The children between 1 and 2.5 years of age were the most difficult to make relaxed and co-operative.

Strengths and limitations of this study: all the children were treated at the same clinic and mainly by the same physician, making treatment uniform, which is a strength. On the contrary, the study only shows the results from one specific hospital. Seven children (10 clubfeet) did not have the first US before the first casting and the degree of deviation from the normal is, therefore, likely to be somewhat underestimated in the average values at the first measurement.

Not all feet could be examined with US at the predetermined ages. Some measurements could not be performed at all examinations (mainly the lateral projections). This small amount of missing data did not appear to influence the overall results in the 4 year-olds, as seen in Figures 7–11.

In the clubfoot cohort, both feet were included in bilateral cases. This may be a potential limitation of the findings^39^ and a sensitivity analysis was therefore performed as a separate calculation with only one foot included in bilateral cases to identify the correlation between the number of casts needed to correct the deformities and the variables of MM-N distance, T-Tang-N distance, and T-N angle. The difference in correlation compared with all clubfeet included was ≤ 10% lower for the MM-N and ≤ 10% higher for the T-Tang-N distance and T-N angle.

Our experience is that the dynamic US findings that reveal the position and mobility of the navicular relative to the talus are most useful to the clinician. The dynamic US can be a valuable aid for learning and teaching the correct manipulation technique.

## Conclusion

US can be used to evaluate the initial degree of deformity in clubfeet and to follow the progress of the treatment and growth of the osseocartilaginous structures and soft tissue. There was a moderate correlation between the US variables of MM-N, T-Tang-N, and T-N angle and the number of casts needed to correct the deformities.

The US showed a clear difference between the clubfeet and the controls during the first four years of life. Even after the correction phase, the MM-N was shorter, the T-Tang-N and T-N angle were larger, and the mean ROM in the T-N joint was approximately 20° less in clubfeet than in controls.

Although it was not possible to define specific limit values as benchmarks in the treatment, dynamic US can provide valuable support in the decision-making process when complementary treatment may be needed.

## Supplementary Material

Supplemental Video 1

Supplementary material

Supplementary material

Supplementary material

Supplementary material

Supplementary material
