# Supplementary material for: A four-year clinical and sonographic longitudinal follow-up of clubfeet treated according to Ponseti with normal references
Source: J Child Orthop. 2023 May 17;17(3):212–23. doi: 10.1177/18632521231172548 (PMC10242368; doi:10.1177/18632521231172548)
Supplement: Supplementary material [file sj-docx-1-cho-10.1177_18632521231172548.docx]

**Video 1.** Dynamic ultrasonography showing the movement in the talo-navicular joint during Ponseti manipulation in a normal foot and in a clubfoot when the child is relaxed and when the child is straining his posterior tibial muscle. Video can be found alongside the online version of this article.
