# Supplementary material for: A four-year clinical and sonographic longitudinal follow-up of clubfeet treated according to Ponseti with normal references
Source: J Child Orthop. 2023 May 17;17(3):212–23. doi: 10.1177/18632521231172548 (PMC10242368; doi:10.1177/18632521231172548)
Supplement: Supplementary material [file sj-docx-2-cho-10.1177_18632521231172548.docx]

**Table 2.** The clubfeet are sorted by age at the start of the plaster treatment.
Course of treatment: casting, orthosis treatment, recurrences, surgical procedures up to the age of eight years
ATT = percutaneous Achilles tendon tenotomy
FAO = Foot abduction orthosis
KAFO = Knee-ankle-foot orthosis
AFO = Ankle-foot orthosis
