# Supplementary material for: A four-year clinical and sonographic longitudinal follow-up of clubfeet treated according to Ponseti with normal references
Source: J Child Orthop. 2023 May 17;17(3):212–23. doi: 10.1177/18632521231172548 (PMC10242368; doi:10.1177/18632521231172548)
Supplement: Supplementary material [file sj-docx-4-cho-10.1177_18632521231172548.docx]

**Table 3.** Valid and missing values for measurements on ultrasound images.
Image missing = a single image is missing, e.g. the child did not co-operate.
Poor image quality = the specific variable could not be measured due to poor image quality. Investigation not performed = a single investigation is missing, or the family (controls) discontinued participating in the study.

STT = Soft tissue thickness
MM-N = Medial malleolus-navicular distance
T-tang-N = Talar-Tangent-Navicular distance
T-N angle = Talo-Navicular angle
Position of the navicular = position of the navicular in relation to the talus in the sagittal plane (normal, plantar, or dorsal)
C-C = Calcaneo-Cuboid distance and angle respectively
Tib. phys.-TCJ = the Tibial physis-Talo Calcaneal Joint distance

At the first examination, the rate of missing images in adduction is high because many clubfeet were already maximally adducted and further adduction was not possible. It was usually also not possible to dorsiflex the foot and, as a result, the frequency of missing Tib. phys.-TCJ in dorsiflexion is high at the first US.
Two controls (four feet) discontinued after 1.5 years, three (6 feet) after three years and two (4 feet) after 3.5 years, so more investigations are therefore missing in the older age groups. The frequency of missing images was high for the lateral coronal projection in the adducted position (it is difficult to obtain perfect images on a convex surface).
